# Tropical tree cover in a heterogeneous environment: A reaction-diffusion model

**DOI:** 10.1371/journal.pone.0218151

**Published:** 2019-06-27

**Authors:** Bert Wuyts, Alan R. Champneys, Nicolas Verschueren, Jo I. House

**Affiliations:** 1 College of Engineering, Mathematics and Physical Sciences, University of Exeter, Exeter, United Kingdom; 2 Bristol Centre for Complexity Sciences, University of Bristol, Bristol, United Kingdom; 3 Applied Nonlinear Mathematics, University of Bristol, Bristol, United Kingdom; 4 School of Geography, University of Bristol, Bristol, United Kingdom; University of Leeds, UNITED KINGDOM

## Abstract

Observed bimodal tree cover distributions at particular environmental conditions and theoretical models indicate that some areas in the tropics can be in either of the alternative stable vegetation states forest or savanna. However, when including spatial interaction in nonspatial differential equation models of a bistable quantity, only the state with the lowest potential energy remains stable. Our recent reaction-diffusion model of Amazonian tree cover confirmed this and was able to reproduce the observed spatial distribution of forest versus savanna satisfactorily when forced by heterogeneous environmental and anthropogenic variables, even though bistability was underestimated. These conclusions were solely based on simulation results for one set of parameters. Here, we perform an analytical and numerical analysis of the model. We derive the Maxwell point (MP) of the homogeneous reaction-diffusion equation without savanna trees as a function of rainfall and human impact and show that the front between forest and nonforest settles at this point as long as savanna tree cover near the front remains sufficiently low. For parameters resulting in higher savanna tree cover near the front, we also find irregular forest-savanna cycles and woodland-savanna bistability, which can both explain the remaining observed bimodality.

## Introduction

First analyses of the satellite-derived MODIS Vegetation Continuous Fields (VCF) tree cover product [1] found strong evidence for the bistability hypothesis [2, 3]. They did this by showing that tropical tree cover data are multimodal at intermediate rainfall values, i.e. they have multiple maxima in their empirical probability distribution function. When taking the plausible assumption that more frequently observed tree cover values are more stable, such multimodality implies multistability. [3] found forest-savanna bistability, from the observation that the tree cover data has a bimodal distribution in a rainfall range of intermediate rainfall, with as modes savanna (about 20% tree cover) and forest (about 80% tree cover). Similarly, [2] found forest-savanna-treeless tristability, with an extra treeless state (about 0%). The treeless state was not found by [3], most likely because they excluded areas with bare soil. A scatterplot of tree cover versus rainfall revealed how the stability of the states depends on rainfall. In such a scatterplot, the modes—stable states according to the dynamical interpretation—show up as regions with high point density. With increasing mean annual rainfall, the inferred probability of being in a higher tree cover mode increases. Hence it was concluded that rainfall can be seen as the bifurcation parameter in a dynamical system with a hysteresis loop. From here, we restrict our focus to forest-savanna bistability.

If the bistability model is valid, the low density regions between the modes indicate instability due to positive feedbacks. To explore the potential mechanisms driving the positive feedback between savanna and forest and to check whether there are additional forcing variables, [3] set up a nonlinear statistical model of tree cover with as predictors mean annual rainfall, dry season length, soil sand content and fire occurrence. They found that both savanna and forest can exist in a regime with mild seasonality (<7 dry months) and intermediate rainfall (1000-2500mm/y). In this regime, forest occurrence is highly predictable from recent fire occurrence, suggesting that fire is an important factor that can explain the positive feedback between the savanna and forest states. The hypothesized mechanism in savannas involves a feedback between grassy cover and fire spread. Fire spread requires a spatially well-connected grassy fuel layer that occurs only below a certain tree cover threshold; below this threshold, fire spread opens up the canopy more, promoting yet better fire spread. Such a mechanism is consistent with previous theoretical and empirical research [4]. The existence of bistability implies that shocks such as forest clearance or drought could lead to a dramatic increase of fire occurrence and tip an area of forest into a savanna state. This area of savanna would then remain locked until large enough increases of rainfall or release of human pressures allow forests to grow back faster than they are lost by intermittent fires.

However, because the empirical studies that support the bistability hypothesis [2, 3] only rely on spatial data, bimodality could be a result of spatially heterogeneous confounding factors, such as climate, plant physiology, soils and human impact. [5–7]. Indeed, in our recent work [8], we showed that, at least in the Amazon region, much of the bimodality is most likely not a consequence of bistability but of spatial heterogeneity due to factors other than rainfall, including rainfall seasonality, soils and human impact. Nonetheless, some bimodality remained in the data, which might still indicate existence of bistability, albeit on smaller scales than claimed previously. One earlier empirical study [9] explored the possibility of more limited bistability than initially inferred. That they still found wide bistability ranges is most likely because they only considered the separate instead of the joint effect of rainfall and seasonality and because they controlled for fewer confounding factors.

Models of tropical tree cover bistability have remained nonspatial [7, 10, 11]. However, interaction between patches is known to be important in tropical forests and savannas, via processes such as seed dispersal, fire spread and water recycling. When allowing spatial interaction under the form of diffusion in single-species reaction-diffusion models with a bistable reaction term, hysteresis and bimodality disappear; instead, there is an environmentally determined point that separates both states [12–14]. Only under the environmental conditions at this point, coined the Maxwell point (MP), can both states coexist. The MP is a well-understood concept in phase transitions theory [15], used in e.g. materials science, plasma physics and mathematical biology. In such applications, it is the point of external conditions (e.g. pressure or temperature) where two separate equilibrium phases of the considered system have the same free energy. Away from the MP, there is always one state that has lower free energy. If the system is spatially homogeneous, perturbations (either diffusion or stochastic effects) will cause invasion fronts by which the state with the lowest free energy will perpetuate throughout the domain. When there is a gradient of external conditions, the front between the stable steady states pins (i.e. it settles) at the MP [8, 12]. This is exactly what we found in our recently developed spatiotemporal model for Amazonian tree cover [8], which consists of a system of equations for several vegetation cover types, including forest and savanna tree cover. While the model without diffusion produces bistability between tree cover states, the spatial model did not produce bistability, but a sharp forest-savanna front, being a function of mean annual rainfall, rainfall seasonality, soils and human impact. Taken together with the limited bimodality in the Amazonian data, this suggests that Amazonian tree cover dynamics can be modeled reasonably well with a single reaction-diffusion equation exposed to heterogeneous external conditions. Nonetheless, the limited amount of remaining bimodality in the data indicates that global bistability, i.e. bistability despite spatial interaction, may still play a role. Alternatively, bimodality can also have arisen from endogenously generated cyclic behavior [10, 16], with cycle periods up to centuries or millennia, posing a real challenge to falsification of the model [16], not least because climatic forcing changes on the same time scales.

Here, we present an analysis of our reaction-diffusion model of tropical tree cover first used in the simulations of [8]. We did not include noise terms as noise was treated extensively in [16]. This model is an expansion of the nonspatial bistability model by [10] through inclusion of spatial effects (diffusion and heterogeneity) and human intervention. In this paper, we refer to the model without savanna trees [*S*, *T* = 0; *F* ≠ 0 in (1)] as the forest model and to the full model with savanna trees [*S*, *T*, *F* ≠ 0 in (1)] as the forest-savanna model. We focus in this work on the analytical derivation of the MP in the homogeneous forest model and its comparison to the front location in the heterogeneous forest model and to simulation results of the heterogeneous forest and forest-savana models. We will show that the MP of the homogeneous forest model is a good predictor of the front between forest and nonforest in the heterogeneous forest-savanna model when savanna tree presence is low. With increasing savanna tree presence, the MP becomes decreasingly accurate at predicting the front. In this regime, savanna-woodland bistability and forest-savanna cycles occur, as shown earlier by [16]. We further show that in the spatial model, the savanna-woodland bistability persists and the forest-savanna cycles can turn irregular.

## Methods

### Forest-savanna model

The full system of partial differential equations representing cover types as a function of space and time, hereafter referred to as the forest-savanna model, can be written as

$$\begin{array}{ccc} \partial_{t}S & = & R_{s}\left(1-S-T-F\right)T-Q_{0}\left[1-h\Phi \left(T,F\right)\right]S-M_{S}S-R_{F}SF+D_{S}\nabla^{2}S,\\ \partial_{t}T & = & Q_{0}\left[1-h\Phi \left(T,F\right)\right]S-M_{T}T-R_{F}TF,\\ \partial_{t}F & = & R_{F}\left(1-F\right)F-b\Phi \left(T,F\right)F-M_{F}F-CF+D_{F}\nabla^{2}F, \end{array}$$ (1)

where

$$\begin{array}{ccc} \Phi (T,F) & = & \frac{\tau^{-1}Y_{c}^{4}}{Y_{c}^{4}+{\left(T+F\right)}^{4}}, \end{array}$$ (2)

and *S* is savanna sapling cover, *T* savanna adult tree cover, *F* forest tree cover, and Φ fraction of area burnt. This model can be obtained by starting from the model of [10] and adding diffusion terms and human impact. *R*_*Y*_, *M*_*Y*_ are growth and mortality rates for *Y* ∈ {*S*, *T*, *F*}. *Y*_*c*_ is the critical value below which fire spread occurs and *τ* the maximum fire return time. *Q*_0_(1 − *h*Φ) is the recruitment rate of savanna saplings into adult savanna trees; a linearly decreasing function of burnt area fraction Φ. *b* is the sensitivity of forest tree cover to fire, which we choose to be constant here. The forest removal rate *C* is a function of distance from human cultivation *z*, or *C* = *C*(*z*). Φ is burnt area fraction, which is a monotonic decreasing and sigmoid-shaped function of nonherbaceous cover 1 − *G* − *S* = *T* + *F*.

We show a systematic way for deriving the model (1) in Supporting Information, Section Model construction. In our previous treatment, we included spatial heterogeneity by letting *R*_*Y*_, *M*_*Y*_ and *Y*_*c*_ be functions of natural environmental forcing variables, such as climate and soils (S1 Table), which in turn depend on space. In this work, we strive to make mathematical analysis as simple as possible, while keeping the model’s essential features. Therefore, we keep rainfall seasonality and soils fixed at their average values, leading to parameters that are only a function of mean annual rainfall *P* or distance to human cultivation *z*. The resulting simplified functional forms and parameter values are shown in Table 1. By assuming that growth rate saturates to a constant maximum *r*_*Y*_ and mortality stabilizes to a constant minimum *m*_0,*Y*_ where water limitation is less severe, we have chosen

$$\begin{array}{ccc} R_{Y}\left(P\right) & = & \max[0,r_{Y}\left(1-e^{-k_{R_{Y}}P+a_{R_{Y}}}\right)],\\ M_{Y}\left(P\right) & = & m_{o,Y}+e^{-k_{M_{Y}}P+a_{M_{Y}}}, \end{array}$$

where for *R*_*Y*_, *Y* ∈ {*S*, *F*} and for *M*_*Y*_, *Y* ∈ {*S*, *T*, *F*}. *k*_*i*_ controls the steepness of the functions and *a*_*i*_ the horizontal position on the *P* axis. Finally, we took

$$\begin{array}{c} Y_{c}\left(P\right)=\text{max}\left[0,Y_{c,0}+k_{c}P\right], \end{array}$$

where *Y*_*c*,0_ > 0 and *k*_*c*_ < 0. *Y*_*c*_(*P*) captures the assumed decreasing percolation threshold (critical value of *T* + *F*) with rainfall. In drier environments, the effective connectivity between areas in space is higher, leading to a higher value of tree cover where fire spread becomes important.

**Table 1:** Model parameters and functional forms of the forest-savanna model when fixing rainfall seasonality and soils at their average (1). These were obtained by filling in the average for rainfall seasonality and soils in the equations of S1 Table.

process and equation	parameter	value	units
cover expansion rate $R_{Y}\left(P\right)=\max\left[0,r_{Y}\left(1-e^{-k_{R_{Y}}P+a_{R_{Y}}}\right)\right]$	*r*_*S*_, *r*_*F*_	0.09,0.20	y^−1^
	$k_{R_{S}},k_{R_{F}}$	0.005,0.003	mm^−1^
	$a_{R_{S}},a_{R_{F}}$	0.25,1.54	-
cover reduction rate by drought $M_{Y}\left(P\right)=m_{Y,o}+e^{-k_{M_{Y}}P+a_{M_{Y}}}$	*m*_*S*,*o*_ = *m*_*T*,*o*_, *m*_*F*,*o*_	0.023,0.041	y^−1^
	$a_{M_{S}}=a_{M_{T}},a_{M_{F}}$	-,-2.15	-
	$k_{M_{S}}=k_{M_{T}},k_{M_{F}}$	0.008,0.008	mm^−1^
savanna tree cover recruitment rate *Q*(Φ) = *Q*_0_(1 − *h*Φ)	*Q*_0_, *h*	0.04,0.85	y^−1^,-
burnt area fraction $\Phi \left(T,F;P\right)=\frac{1}{\tau }\frac{Y_{c}^{n}}{Y_{c}^{n}+{\left(T+F\right)}^{n}},$	*τ*, *n*	2.7,4	y,-
critical cover value for fire spread *Y*_*c*_(*P*) = max[0, *Y*_*c*,0_ + *k*_*c*_*P*]	*Y*_*c*,0_	0.56	-
	*k*_*c*_	-1.43e-04	mm^−1^
forest cover fire sensitivity deforestation rate *C*(*z*) = *ce*^−*k*_*C*_*z*^	*b*	0.46	-
	*c*, *k*_*C*_	0.092,0.0015	-,m^−1^
diffusion coefficient of *S*, *F*	*D*_*S*_, *D*_*F*_	0.2,0.1	km^2^y^−1^

To introduce spatial heterogeneity, and having already chosen how *R*_*Y*_, *M*_*Y*_ and *Y*_*c*_ depend on *P*, we still have to choose how *P* depends on space. We do this by taking

$$\begin{array}{c} P(x)=x. \end{array}$$ (3)

The resulting rainfall gradient of 1mm/km lies in the range of what can be expected in the tropics.

### Forest model (S,T = 0)

We now set up the spatial model of forest cover (and its complement 1 − *T*, grass cover). This is done by setting *S* = *T* = 0 in (1), leading to

$$\begin{array}{c} \partial_{t}F=R_{F}\left(P\right)F\left(1-F\right)-M_{F}\left(P\right)F-bF\Phi \left(F\right)-C\left(z\right)F+D_{F}\nabla^{2}F. \end{array}$$ (4)

It will be helpful in the analysis that follows to produce a nondimensional version of this model. We first take *u* = *F* and rescale *t* → *bt*/*τ*. We take as nondimensional constants (see Table 1),

$$\begin{array}{c} \rho =\frac{r_{F}}{b}\tau ,\mu =\frac{e^{a_{M_{Y}}}}{b}\tau ,\mu_{0}=\frac{m_{F,o}}{b}\tau ,\gamma =\frac{c}{b}\tau ,\delta_{F}=\frac{D_{F}}{b}\tau , \end{array}$$

and replace $\kappa_{r}=k_{R_{F}},\kappa_{m}=k_{M_{F}},a=a_{R_{Y}},u_{c,0}=Y_{c,0}$ for lighter notation. We further take as nondimensional functions

$$\begin{array}{ccc} r(P) & = & 1-e^{-\kappa_{r}P+a},\\ m(P) & = & e^{-\kappa_{m}P},\\ f(u;P) & = & \frac{u_{c}{\left(P\right)}^{4}}{u_{c}{\left(P\right)}^{4}+u{}^{4}},\\ u_{c}\left(P\right) & = & \max[0,u_{c,0}+k_{c}P],\\ c(z) & = & e^{-k_{C}z}. \end{array}$$

When putting everything together, the following dimensionless form of the PDE is obtained,

$$\begin{array}{c} \partial_{t}u=\rho r\left(P\right)\left(1-u\right)u-\mu m\left(P\right)u-uf\left(u;P\right)-\gamma c\left(z\right)u-\mu_{0}u+\delta_{F}\nabla^{2}u. \end{array}$$

With rescaling $x\to \sqrt{\delta_{F}}x$ we then obtain

$$\begin{array}{c} \partial_{t}u=\rho r\left(P\right)\left(1-u\right)u-\mu m\left(P\right)u-uf\left(u;P\right)-\gamma c\left(z\right)u-\mu_{0}u+\nabla^{2}u. \end{array}$$

When making the further substitutions,

$$\begin{array}{ccc} \alpha (P,z) & = & \rho r\left(P\right)-\mu m\left(P\right)-\gamma c\left(z\right)-\mu_{0},\\ \beta (P) & = & \rho r(P), \end{array}$$

we obtain

$$\begin{array}{c} \partial_{t}u=\alpha \left(P,z\right)u-\beta \left(P\right)u^{2}-uf\left(u;P\right)+\nabla^{2}u. \end{array}$$ (5)

We will show that the front between forest and grassland as a function of the forcing variables can be found analytically. While this model does not include savanna tree cover, we can compare the forest-savanna model with this one to see how the presence of savanna trees affects the results.

### Parameters, simulation and figures

All parameter values have roughly the same values as those in [8]. S1 Table summarizes the parameters and functions used in the model. The forest growth rate can be easily inferred from the data (see Supporting Information Section Forest growth rate). We ran the 1D model in MATLAB [17] with the ode45 algorithm based on Runge-Kutta 4th and 5th order temporal discretization (variable Δt) and central difference spatial discretization (Δ*x* = 0.67), no-flux boundary conditions and random initial conditions. The chosen left and right boundaries are 0km and 3000km.

**Fig 1:**
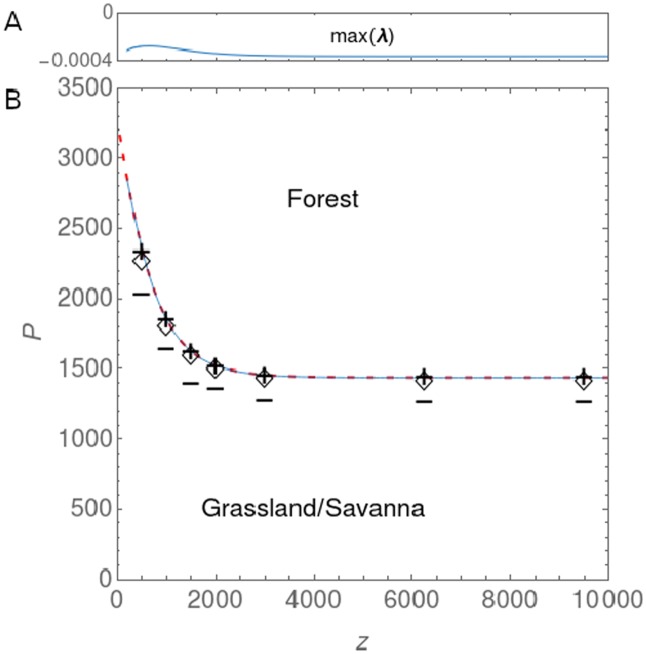
Maxwell point in the homogeneous forest model and pinning rainfall in the heterogeneous models. (A) Maximum eigenvalue of (13) for a range of *z* values in the heterogeneous forest model (i.e. along the solid blue line in panel B). (B) Front between forest and savanna/grassland in (*P*, *z*) space for different models. The dashed red line shows the theoretically derived MP from the homogeneous forest model and the solid blue line the location of the forest front in the heterogeneous forest model obtained by a numerical continuation. Markers show at which rainfall value the front settles in the heterogeneous models for given *z* values: (+) forest model (4), (◊) forest-savanna model (1) with *r*_*S*_ = 0.09 and *Q*_0_ = 0.04, (−) forest-savanna model (1) with *r*_*S*_ = 0.13 and *Q*_0_ = 0.09.

**Fig 2:**
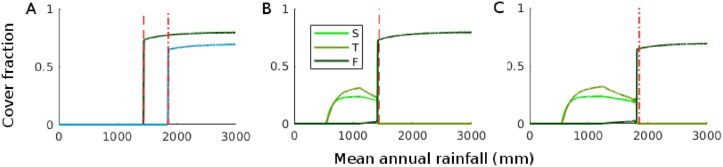
Simulation results and the effect of human impact for the models under low impact of savanna trees (*r*_*S*_ = 0.09, *Q*_0_ = 0.04, *τ* = 2.7). (A) Forest model (4) under natural (green) and impacted conditions (blue, 1km from cultivated areas). (B) Forest-savanna model (1) under natural conditions. (C) Forest-savanna model (1) with human impact (1km from cultivated areas). The red dashed line shows the derived value of the MP in the natural forest model. The red dash-dotted line shows the derived value of the MP in the forest model with human impact. Rainfall can also be seen as a spatial coordinate because the model was forced by heterogeneous rainfall *P*(*x*) = *x*.

**Fig 3:**
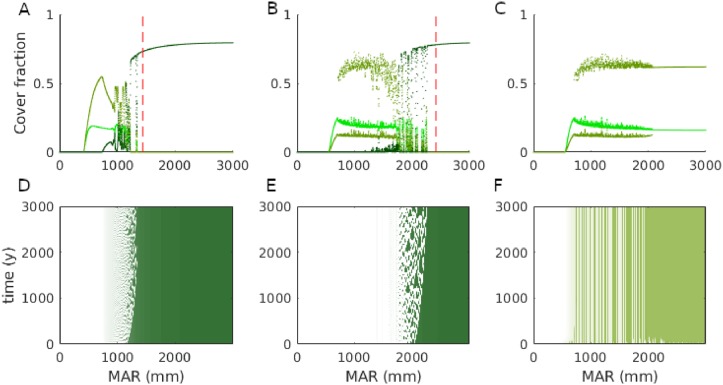
Simulation results of the forest-savanna model (1) with higher savanna sapling growth rate (*r*_*S*_ = 0.13) and: (A,D) higher sapling recruitment into adults (*Q*_0_ = 0.09), (B,E) higher recruitment into adults and lower fire return interval (*Q*_0_ = 0.2, *τ* = 1). (C,E) Same as in (B,E) but without forest trees. The upper panels show cover fraction versus rainfall at the end of the simulation and of all cover types. The lower panels show forest (D,E) or of savanna adult tree cover over the spatial domain (with the location indicated by its rainfall) as a function of time. The MP of the corresponding forest model is shown with the dashed red line. See Fig 1 for legend.

We made two types of figures: a phase plot with the front location in parameter space (Fig 1), and, scatterplots of cover types versus rainfall in the heterogeneous models (Figs 2 and 3). To create the phase plot of the heterogeneous models in Fig 1, we needed to extract the rainfall value at the front from the model output. For the simulations (markers in Fig 1), we did this via a robust curve fitting method, fitting the logistic function (goodness of fit *R*^2^ > .9),

$$\begin{array}{c} F^{*}\left(P\right)=\frac{A}{1+\exp[-k\left(P-P_{f}\right)]}, \end{array}$$ (6)

and extracting *P*_*f*_, with the MATLAB [17] curve fitting tool. In the numerical continuation of the heterogeneous forest model (solid blue line in Fig 1), we did this via

$$\begin{array}{c} P_{f}=\arg\max F_{x}^{*}\left(P\right), \end{array}$$ (7)

where $F_{x}^{*}$ is the spatial derivative of the front solution. We used (6) instead of (7) in the simulations because in the forest-savanna model, savanna species can induce gradients of *F* away from the front. The two methods give the same results when there are no savanna trees (compare + and solid blue line in Fig 1).

The analysis of the homogeneous model almost exclusively required symbolic analysis, which we did with Mathematica [18].

## Results

In the first section below, we derive the MP of the homogeneous forest model. In the second section, the front pinning location in the heterogeneous forest model is derived via a numerical continuation. The third section shows simulation results of the heterogeneous forest and forest-savanna models.

### Maxwell point of the homogeneous forest model

For simplicity we shall consider one spatial dimension, which gives rise to scalar fronts rather than domain boundaries in the form of line fronts. While the approach in 2D is identical once one chooses a direction of propagation of any invasion front, front dynamics will, unlike in 1D, be influenced by front curvature, but this is minimal for the spatial scales considered [8, 19]. Because we first assume forcing to be homogeneous, we can further treat *p* and *z* as parameters. We further also assume that the front width is very small compared to the domain size, such that it is justified to take the domain size as approximately infinite.

When starting from (5), hiding the dependence on *p* and *z*, grouping common factors, and indicating further ∂_*t*_*u* by *u*_*t*_ and ∂^2^*u*/∂*x*^2^ by *u*_*xx*_, we obtain

$$\begin{array}{c} u_{t}=\left[\alpha -\beta u-f\left(u\right)\right]u+u_{xx}. \end{array}$$ (8)

As the nonlinear term causes bistability, we expect traveling front solutions [see e.g. [13, 14, 20]] between the stable steady states of the form *u*(*ξ*) with *ξ* = *x* − *ct* and *c* the wave speed, with boundary conditions *u*(−∞) = *u*_−_ and *u*(∞) = *u*_+_ such that we can rewrite our equation as

$$\begin{array}{c} -cu^{\prime }=\left[\alpha -\beta u-f\left(u\right)\right]u+u^{\prime \prime }, \end{array}$$

where *u*′ = *du*/*dξ* and *u*″ = *d*^2^*u*/*dξ*^2^. When multiplying by *u*′, we obtain

$$\begin{array}{c} -c{\left(u^{\prime }\right)}^{2}=\left[\alpha -\beta u-f\left(u\right)\right]uu^{\prime }+u^{\prime \prime }u^{\prime }. \end{array}$$

Integrating this with respect to *ξ* over the real axis, we further obtain

$$\begin{array}{ccc} -c\int_{-\infty }^{\infty }{\left(u^{\prime }\right)}^{2}d\xi & = & \int_{-\infty }^{\infty }\left[\alpha -\beta u-f\left(u\right)\right]uu^{\prime }d\xi +\int_{-\infty }^{\infty }u^{\prime \prime }u^{\prime }d\xi ,\\ & = & \int_{u_{-}}^{u_{+}}\left[\alpha -\beta u-f\left(u\right)\right]udu-\int_{u_{-}}^{u_{+}}u^{\prime }du^{\prime },\\ & = & \int_{u_{-}}^{u_{+}}\left[\alpha -\beta u-f\left(u\right)\right]udu-{\left[\frac{1}{2}u^{\prime 2}\right]}_{u_{-}}^{u_{+}}. \end{array}$$

As the solution is flat at the boundaries, we have ${\left[\frac{1}{2}u^{\prime 2}\right]}_{u_{-}}^{u_{+}}=0$, such that

$$\begin{array}{c} -c\int_{-\infty }^{\infty }{\left(u^{\prime }\right)}^{2}d\xi =\int_{u_{-}}^{u_{+}}\left[\alpha -\beta u-f\left(u\right)\right]udu. \end{array}$$

As the integrand of the left hand side of this expression is always positive, we have

$$\begin{array}{c} \text{sign}\left(c\right)=\text{-sign}\{\int_{u_{-}}^{u_{+}}\left[\alpha -\beta u-f\left(u\right)\right]udu\}=\text{sign}\left(\Delta V\right), \end{array}$$ (9)

where we have defined,

$$\begin{array}{ccc} \Delta V & \equiv & -\int_{u_{-}}^{u_{+}}\left[\alpha -\beta u-f\left(u\right)\right]udu\\ & = & {\left[-\alpha u^{2}/2+\beta u^{3}/3\right]}_{u_{-}}^{u_{+}}+\int_{u_{-}}^{u_{+}}f\left(u\right)udu. \end{array}$$

Hence, we see that the dynamics can be derived from the potential by

$$\begin{array}{c} u_{t}=-V_{u}+\nabla^{2}u. \end{array}$$

At the MP, the front is stationary, i.e. *c* = 0, such that according to (9),

$$\begin{array}{c} \Delta V={\left[-\alpha u^{2}/2+\beta u^{3}/3\right]}_{u_{-}}^{u_{+}}+\int_{u_{-}}^{u_{+}}f\left(u\right)udu=0. \end{array}$$

This allows calculation of an expression for the MP as a function of the parameters *α* and *β*. These parameters, in turn, are a function of the external forcings of the model.

If we choose *f*(*u*) as in equation S1 in S1 Text with *Y* = *u*,

$$\begin{array}{c} f\left(u\right)=\frac{u_{c}^{4}}{u_{c}^{4}+u{}^{4}}, \end{array}$$ (10)

then ∫ *f*(*u*)*du* can be calculated analytically as

$$\begin{array}{c} \int f\left(u\right)du=\frac{u_{c}^{2}}{2}\text{arctan}\left[(u/u_{c}{)}^{2}\right], \end{array}$$

such that

$$\begin{array}{c} V\left(u\right)=\frac{\beta u^{3}}{3}-\frac{\alpha u^{2}}{2}+\frac{u_{c}^{2}}{2}\text{arctan}\left[(u/u_{c}{)}^{2}\right]. \end{array}$$

Δ*V* = *V*(*u*_+_) − *V*(*u*_−_) can be found analytically if *u*_+_ and *u*_−_ can be found analytically. However, *u*_+_, *u*_−_ can only be found analytically when the (integer) exponent in equation S1 in S1 Text is 1 ≤ *n* ≤ 3. As we chose *n* = 4, this step has to be done numerically. From here, the MP can be calculated by finding the root of Δ*V* as a function of its parameter(s). Also this is only possible numerically. The result of this calculation is shown as the dashed red line in Fig 1. For the parameters shown in Table 1, without human impact, and, at average rainfall seasonality and soils, the MP of the forest model lies at a mean annual rainfall of 1438mm. Areas receiving *P* > 1438mm will experience an invasion of forest while areas receiving *P* < 1438mm will experience loss of forest. When including human impact, forest is only considerably affected by human impact when it is less than *z* ≈ 2km away from agricultural areas.

Without spatial interaction in the forest model, there is a wide range where forest is bistable with grassland (∼1200-3500mm, upper branch and lower zero branch indicated with solid lines in S1 Fig). Hence, we showed here that including spatial interaction causes the bistability range to collapse into one point—the MP. Note that when there are *N* forcing variables, the MP is not a point but a *N* − 1 dimensional surface in phase space. Away from the MP, the only stable state is the one with lowest potential energy *V*. The alternative state with lower potential energy is now metastable. It can persist when: (1) the whole spatial domain is homogeneously in that state, and (2) that this homogeneous state is not sufficiently perturbed. Nonetheless, neither of these conditions are easily met in reality.

### Front pinning in the heterogeneous forest model

When external conditions are heterogeneous, the parameters *p*, *z* and the solutions *u*_+_, *u*_−_ are functions of *x* and the approach in the previous section cannot be used any more. However, one can expect that when the spatial dependence is weak, it can still be used as an approximation. It can then be expected that in the limit of *t* → ∞, areas receiving *P* > *P*_*MP*_ will have forest while areas receiving *P* < *P*_*MP*_ will not have forest, with the front pinned at *P*_*MP*_. The stability of the pinned front solution can be verified via a linear stability analysis. When writing the reaction term of (8) as R[u;P], we have

$$\begin{array}{c} u_{t}=R\left[u\left(x\right);P\left(x\right)\right]+u_{xx}. \end{array}$$ (11)

At the front solution *u* = u*(*x*), we perturb the solution with *δu*(*x*, *t*) ≪ 1 and see how this perturbation grows by substituting u*(*x*) + *δu*(*x*, *t*) and neglecting higher order terms in *δu*,

$$\begin{array}{ccc} {\left[u^{*}\left(x\right)+\delta u\left(x,t\right)\right]}_{t} & = & R\left[u^{*}\left(x\right)+\delta u\left(x,t\right);P\left(x\right)\right]+{\left[u^{*}\left(x\right)+\delta u\left(x,t\right)\right]}_{xx},\\ {\left[\delta u\left(x,t\right)\right]}_{t} & = & R\left[u^{*}\left(x\right);P\left(x\right)\right]+\frac{\partial R}{\partial u}\left[u^{*}\left(x\right),P\left(x\right)\right]\left[\delta u\left(x,t\right)\right]+{\left[u^{*}\left(x\right)+\delta u\left(x,t\right)\right]}_{xx},\\ {\left[\delta u\left(x,t\right)\right]}_{t} & = & \frac{\partial R}{\partial u}\left[u^{*}\left(x\right),P\left(x\right)\right]\left[\delta u\left(x,t\right)\right]+{\left[\delta u\left(x,t\right)\right]}_{xx}, \end{array}$$

where the second step is possible because $R\left(u^{*};P\right)+u_{xx}^{*}=0$ as u* is a solution of (11). Therefore, the front solution is only stable with respect to perturbation when all eigenvalues of the operator,

$$\begin{array}{c} L\left(x\right)=\frac{\partial R}{\partial u}\left[u^{*}\left(x\right),P\left(x\right)\right]+\partial_{xx}, \end{array}$$ (12)

have negative real parts. In our case, it is not possible to obtain the front solution u*(*x*) analytically. Therefore, linear stability can be evaluated numerically, by calculating the eigenvalues of the discretized form of (12), which is the *n* × *n* matrix

$$\begin{array}{c} L=\frac{\partial R}{\partial u}\left(u^{*},P\right)I+\frac{L}{\Delta x^{2}}, \end{array}$$ (13)

where u* = [*u*(*x*_0_), *u*(*x*_1_), …, *u*(*x*_*n*−1_)] and **P** = [*P*(*x*_0_), *P*(*x*_1_), …, *P*(*x*_*n*−1_)] are the discretized front solution and rainfall values as a function of space, with *x*_*k*_ = *x*_0_ + *k*Δ*x*, **L**/Δ*x*^2^ the discretized Laplacian, and **I** the identity matrix. If we define max **v** as the maximum of a vector **v**’s elements and **λ** as the vector with *n* eigenvalues of (13), the condition for stability is hence

$$\begin{array}{c} \text{max}\;ℜ(\lambda )<0, \end{array}$$ (14)

where ℜ indicates that we take the real part. Because the front solution u* depends on all the parameters, L is calculated for only one point in parameter space. To obtain information on the stability of all front solutions in a given parameter range, one needs to obtain the solution for a set of points in that range and evaluate L for each of them. Starting from a known front solution, pseudo-arclength continuation [21–23] allowed us to find other front solutions of (11) in parameter space. To compare the results with those of the previous section, we plot the rainfall value at which the front pins in the heterogeneous equation as a function of *z* (distance from human cultivation). We extracted the location of the front via (7) for each value of *z*. Our analysis shows that the front solution of the heterogeneous forest model (solid blue line in Fig 1) is indistinguishable from the MP of the homogeneous forest model (dashed red line in Fig 1). Moreover, we found that for each value of *z* considered (14) is satisfied (solid red line in Fig 1), indicating that each front solution is a stable steady state, or more specifically, a stable node, as all eigenvalues of (13) are real. It can hence be concluded that, at least for our setup with weak spatial dependence, the front of the heterogeneous forest equation pins at the MP of the homogeneous forest equation.

### Simulation of the heterogeneous models

Here we show steady state profiles of vegetation by the heterogeneously forced models. We remind the reader that the used forcing is a linear relation between distance from the origin and rainfall (3) such that at the chosen left and right boundaries *P*(0km) = 0mm and *P*(3000km) = 3000mm, respectively. Therefore, the x-axis of the plots in Figs 2 and 3 is both distance from the origin in km or mean annual rainfall in mm.

Fig 2 shows the steady states of *F* as a function of rainfall by the forest model (5) and of *S*, *T* and *F* by the forest-savanna model (1) for parameters leading to low savanna tree presence (*r*_*s*_ = 0.09, *Q*_0_ = 0.04, *τ* = 2.7), with and without human impact. Without human impact, all models have their forest front pinned at a rainfall value of about 1400mm [Fig 2A (green), Fig 2B (green)], with forest occurring above and grassland or savanna below this value. Adding human impact results in a shift of the forest front to higher rainfall values (Fig 2A blue versus green; Fig 2C vs 2B). In the forest model, the MP obtained from the analysis of the homogeneous model (Fig 2, dashed lines without human impact and dash-dotted line with human impact) accurately predicts the location of the forest front (Fig 2A). The model with savanna trees (forest-savanna model) has its forest front at slightly lower rainfall values than the model without savanna trees (Fig 2C vs 2B). The rainfall value at which the front pins is indicated by markers in Fig 1 for a wider range of *z* values, confirming the good match [perfect match for the forest model (+) and small bias for the forest-savanna model (◊)] between the rainfall value at which the front pins and the MP of the homogeneous forest model for the parameters chosen here.

Fig 3A shows the cover types versus rainfall when we choose parameters leading to higher savanna tree cover (*r*_*s*_ = 0.13, *Q*_0_ = 0.09). As before, there is forest on the wet side and savanna on the dry side of the x axis. However, now adult savanna trees reach higher cover values and there is a larger difference between the MP and the location of the front (see − markers in Fig 1 for a wider range of *z* values). The MP becomes decreasingly accurate as predictor of the forest front with increasing savanna tree cover (Fig 3B versus Figs 3A and 2D). Moreover, beyond the point where savanna cover decreases, there is a range of rainfall values below the forest front where forest and savanna tree cover show high variation due to irregular oscillations of forest and savanna tree cover (Fig 3D). Fig 3B shows that when savanna tree recruitment is increased further (*Q*_0_ = 0.2) and when also the fire return interval is decreased (*τ* = 1), savanna tree cover becomes bistable below a rainfall of about 1000mm and the range of rainfall with forest-savanna cycles widens. We will further refer to the low savanna tree cover state as the savanna state and to the high savanna tree cover state the woodland state. Note that the savanna tree cover bistability also occurs (for the same parameters) without forest trees (Fig 3C and 3F), but up to a rainfall of about 2500mm.

**Fig 4:**
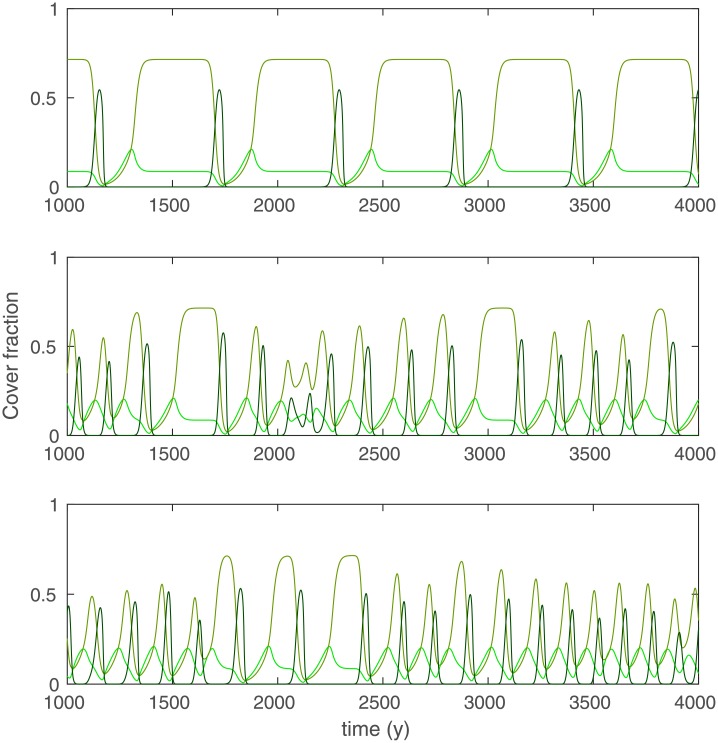
Cycles in the forest-savanna model with high savanna tree presence (*r*_*S*_ = 0.13, *Q*_0_ = 0.2 and *τ* = 1): (A) nonspatial model with *P* = 1500mm, (B) spatially homogeneous model with *P* = 1500mm, (C) Spatially heterogeneous model at the point where *P* = 1500mm. See Fig 2 for legend.

In Fig 4, we show the forest-savanna cycles in more detail. During the cycles, forest tree cover lags behind savanna tree cover. The changes between states occur over decades, but the periods of stability between the transitions can persist for several centuries (or longer, depending on the parameters). The nonspatial system only produces a regular cycle (Fig 4A) while the spatially homogeneous system with diffusion (Fig 4B) has irregular cycles. The spatially heterogeneous system has similar irregular cycles (Fig 4C). The irregularity of these cycles can hence be induced by diffusion alone.

## Discussion

In this paper, we have provided a first analytical and numerical analysis of our spatially heterogeneous reaction-diffusion model of tropical tree cover. We have treated this model before with a more realistic set-up [8] (in 2D, with noise and forced by observed climate, soil and human impact), but we formulated it here in an as simple as possible form (in 1D, deterministic and forced by linear rainfall) for easier mathematical analysis. The heterogeneity was captured with the relation (3), such that low x values represent dry and high x values represent wet areas. From the homogeneous system without savanna trees/saplings [*S* = *T* = 0, (5)], a Maxwell point was derived. We showed via a numerical continuation and linear stability analysis of the spatially heterogeneous forest model that this MP is still of use for the spatially heterogeneous case because here, it is the parameter value at which the forest front pins. The MP of the homogeneous forest model and the rainfall value at which the forest model’s front pins as a function of external parameters (the dashed red line and the solid blue line in Fig 1 respectively) are indistinguishable and have the same shape as what was obtained in [8] by simulation. Existence of a MP in reaction-diffusion equation with a bistable reaction term [13, 14, 20] and pinning under heterogeneity [12] is consistent with previous work. For parameters that lead to low cover of savanna trees, the MP of (5) is also a good predictor of the forest-savanna model’s forest front [*S*, *T* ≠ 0, (1)] (Fig 2C–2F). This is because the effect of savanna trees on forest trees, mediated by burnt area [see (1)], remains negligible when savanna tree cover near the forest front stays below the threshold where fire spread is inhibited, i.e. *T* < *Y*_*c*_. Choosing parameters such that savanna tree cover near the forest front exceeds this threshold (*T* ≳ *Y*_*c*_) makes the forest front shift away from the MP of (5), towards drier areas (Fig 3A and 3B). In this regime where savanna tree cover affects forest tree cover, we also found forest-savanna cycles and savanna-woodland bistability, which both can lead to bimodal tree cover distributions under the same external forcings. These cycles are consistent with the existence of Hopf bifurcations in the nonspatial system [16] above a certain value of the parameters equivalent to *P* and *r*_*s*_. For an explanation of the physical mechanism behind the cycles, we refer to [16]. We found that the cycles can turn irregular by diffusion. That the irregular cycles are produced endogenously suggests that close to the forest front, sudden and unpredictable loss of forest can occur without climatic or anthropogenic perturbations. We speculate that the irregularity is due to spatiotemporal chaos, which is known to occur in the wake of traveling fronts [24, 25]. To prove this, it would need to be shown additionally that the cycles produced by the deterministic system are truly aperiodic and that there is sensitivity to initial conditions [26]. We further showed via simulation that bistability of a savanna and a woodland state can arise in the savanna model (i.e. the model without forest trees) under a regime of high sapling recruitment and high fire occurrence (Fig 3C and 3F). When introducing forest trees (under the same conditions), the savanna-woodland bistability does not survive at higher rainfall, due to competition between savanna and forest trees (Fig 3B). Instead, the irregular cycle discussed above appears. Where it is too dry for forest, savanna tree cover bistability does survive. To obtain a complete picture of the behavior of the spatial model and how it differs from the nonspatial model, its bifurcation diagrams need to be made. A step towards increased realism is then the consideration of two spatial dimensions instead of one, with a further step towards increased realism being the verification of how this diagram is affected by spatial heterogeneity.

Taking our results reported here together with the simulation results in our previous work [8] and other recent work [16], the forest-savanna model can produce bimodal tree cover distributions in a range of external parameters due to: (i) bistability between savanna and woodland, (ii) existence of forest-savanna cycles, (iii) spatial heterogeneity of forcings other than rainfall. Fitting our model for separate regions to data in empirically justified parameter ranges might reveal differences between different regions or suggest which model components are not adequately captured. That much of the tree cover bimodality in the Amazon region can be attributed to spatial heterogeneity, leaving little remaining bimodality [8], indicates on one hand that bistability and cyclic behavior play at most a small role in Amazonia. Nonetheless, dry forests in Amazonia and elsewhere might still exist as an alternative state to moist forest and/or savanna. In Africa, where there exist large areas of high tree cover savannas [27] and where fire occurrence is higher [11], bistability and cyclic behavior can be expected to play a larger role. A possibility other than the ones hitherto mentioned is that the observed bimodality is an artifact, resulting from data algorithms [28] or preprocessing methods [29]. Therefore, the multistability hypothesis should be tested on tree cover data produced with methods that are less likely to generate such artifacts.

Finally, there exist other types of feedbacks than assumed here and which can induce multistability. These include feedbacks between soil fertility and vegetation [30], rainfall and vegetation [31–34], and, herbivore presence and vegetation. As all existing feedbacks may interact on various scales [35], there is no doubt that tropical vegetation is not just complex but also complicated. Nevertheless, the insight from complexity science that complicated dynamics can emerge from simple rules suggests that they might be less complicated than we currently think. In the search for such simple rules, spatiotemporal conceptual models like the one developed here will be indispensable. On the other hand, even if the rules turn out to be simpler than expected initially, their resulting dynamics may only be captured realistically when they are implemented in models that are sufficiently individual based.

## Supporting information

S1 TableModel parameters of the forest-savanna model equation S3 in S1 Text.**A** = **A**(**x**). The components of are: *A*_1_ = *P* (mean annual rainfall), *A*_2_ = *M* (Markham’s seasonality index), $A_{3}=\pi -\bar{\pi }$ (edaphic forest suitability). *π* captures the effect of soils on forest occurrence and is taken from [8], i.e. *A*_3_ = 0.00238*φ*_*s*_ − 0.188*φ*_*c*_ − 5.99*ρ* − 0.183*φ*_*c*_*ρ* + 6.39, where *ρ* is topsoil bulk density, *φ*_*s*_ topsoil sand fraction, and *φ*_*c*_ topsoil clay fraction. The components of the vectors ***k***_*i*_ multiply the components of **A**. If a component is indicated as ‘-’, the considered equation is not a function of the corresponding component of **A**.(PDF)

S1 Fig Homogeneous steady states (HSS) of forest cover (*F*) in the forest model without human impact [*C*(*z*) = 0 in (4)] as a function of mean annual rainfall (*P*) for average soils and with rainfall seasonality (*MSI*) as indicated in the legend. Stable states are indicated with solid lines and unstable steady states with dashed lines. HSS are steady states of the nonspatial model (*δ* = *D*_*F*_ = 0). These plots were obtained by finding the roots of the reaction term in (4). The stable branches (solid) are metastable states in the spatial model—they can persist if the whole domain is in the same state and if they are not exposed to perturbations larger than a small threshold.
(EPS)

S2 Fig Recovery time of undisturbed moist forest as a function of parameter *r*_*F*_ when taking F* = 0.8 and when the initial forest cover *F*_0_ = 0.01, based on equation S5 in S1 Text. (EPS)

S1 Text Model construction. Forest growth rate *r*_*F*_.
(PDF)
