# Supplementary material for: Tropical tree cover in a heterogeneous environment: A reaction-diffusion model
Source: PLoS One. 2019 Jun 27;14(6):e0218151. doi: 10.1371/journal.pone.0218151 (PMC6597153; doi:10.1371/journal.pone.0218151)
Supplement: S1 Table — A = A(x). The components of are: A1 = P (mean annual rainfall), A2 = M (Markham’s seasonality index), A3=π−π¯ (edaphic forest suitability). π captures the effect of soils on forest occurrence and is taken from [8], i.e. A3 = 0.00238φs − 0.188φc − 5.99ρ − 0.183φcρ + 6.39, where ρ is topsoil bulk density, φs topsoil sand fraction, and φc topsoil clay fraction. The components of the vectors ki multiply the components of A. If a component is indicated as ‘-’, the considered equation is not a function of the corresponding component of A. (PDF) [file pone.0218151.s001.pdf]

process and equation	value	parameter	units
cover expansion rate	0.09,0.20	r_S, r_F	y^{-1}
$R_Y(\mathbf{A}) = \max[0, r_Y(1 - e^{-\mathbf{k_{R_Y}} \cdot \mathbf{A} + a_{R_Y}})]$	(0.005,-,-)	$\mathbf{k}_{R_S}$	$(\mathrm{mm}^{-1}$,-,-)
	(0.003,3.26,-)	$\mathbf{k}_{R_F}$	$(\mathrm{mm}^{-1}$,-,-)
	0.25,0.196	a_{R_S}, a_{R_F}	-
cover reduction rate by drought	0.023,0.041	$m_{S,o} = m_{T,o}, m_{F,o}$	y^{-1}
$M_Y(\mathbf{A}) = m_{Y,o} + e^{-\mathbf{k}_{M_Y} \cdot \mathbf{A} + a_{M_Y}}$	-,-2.15	$a_{M_S} = a_{M_T}, a_{M_F}$	-
	(0.008,-,-)	$\mathbf{k}_{M_S} = \mathbf{k}_{M_T}$	$(\mathrm{mm}^{-1}$,-,-)
	(0.008,-4.66,1.5)	$\mathbf{k}_{M_F}$	$(\mathrm{mm}^{-1}$,-,-)
savanna tree cover recruitment rate $Q(\Phi) = Q_0(1 - h\Phi)$	0.04,0.85	Q_0, h	y^{-1},-
local burnt area fraction $\Phi(T, F; \mathbf{A}) = \frac{1}{\tau} \frac{Y_c(\mathbf{A})^n}{Y_c(\mathbf{A})^n + (T+F)^n}$,	2.7,4	τ, n	y^{-1},-
with: $Y_c(\mathbf{A}) = \max[0, Y_{c,0} + \mathbf{k}_c . \mathbf{A}]$	0.484	$Y_{c,0}$	-
	(-1.43e-04,.2,-.1)	$\mathbf{k}_c$	$(\mathrm{mm}^{-1}$,-,-)
forest cover fire sensitivity	0.46	b	-
deforestation rate $C(z) = ce^{-k_C z}$	0.092,0.0015	c, k_C	-,m^{-1}
diffusion coefficient of F, S	0.1,0.2	D_F, D_S	$\mathrm{km}^2\mathrm{y}^{-1}$
