# Supplementary material for: Tropical tree cover in a heterogeneous environment: A reaction-diffusion model
Source: PLoS One. 2019 Jun 27;14(6):e0218151. doi: 10.1371/journal.pone.0218151 (PMC6597153; doi:10.1371/journal.pone.0218151)
Supplement: S1 Text — Forest growth rate rF. (PDF) [file pone.0218151.s004.pdf]

Tropical Tree Cover in a Heterogeneous Environment: A Reaction-diffusion Model (*Supporting Information)*

Bert Wuyts[1,2,3,*], Alan R. Champneys[3], Nicolas Verschueren[1,3], Jo I. House[4],

1 College of Engineering, Mathematics and Physical Sciences, University of Exeter, Exeter 44QF, United Kingdom

2 Bristol Centre for Complexity Sciences, University of Bristol, Bristol BS28BB, United Kingdom

3 Applied Nonlinear Mathematics, University of Bristol, Bristol BS81UB, United Kingdom

4 School of Geography, University of Bristol, Bristol BS81SS, United Kingdom

[*]Corresponding author; e-mail: b.wuyts@ex.ac.uk

Model construction

General overview

The model in space and time can be written as a system of stochastic partial differential equations of the reaction-diffusion type. In ecological context, one describes the dynamics of a species density as the sum of a reaction term, representing local demography, and a diffusion term, representing migration of the species through space from areas with high to areas with low density. Our model can be most compactly expressed in vector form

$$\partial_t \mathbf{Y} \quad = \quad \mathbf{J}(\mathbf{Y};\mathbf{A}) + \mathbf{D}\nabla^2\mathbf{Y},$$

where $\mathbf{Y} = (S, T, F, G)$ with S representing savanna sapling cover density, T savanna tree cover density, F forest tree cover density, and G grass cover density. $\mathbf{A}$ is a vector of exogenous environmental variables or parameters that force the system, such as mean rainfall, rainfall seasonality and soils [8]. These forcing variables are in general all heterogeneous in space, but in the main text we kept rainfall seasonality and soils constant, and took mean annual rainfall as the only heterogeneous forcing. $\mathbf{J}$ is a vector of reaction terms representing local population dynamics involving both gains and losses and contains nonlinear terms in both $\mathbf{Y}$ and $\mathbf{A}$. Note that one other forcing variable is contained in $\mathbf{J}$, being human impact. With regards to qualitative dynamics and steady state distributions, the particular choices of the functional forms of $\mathbf{J}$ are arbitrary to some extent. As long as we choose the right shape, the phase portrait should be topologically equivalent to the true functional form. $\mathbf{D}$ is a diagonal matrix with diffusion constants. We take the forcing variables as constant in time. This is done by replacing $\mathbf{A}(\mathbf{x}, t)$ by its long-term mean, which is only a function of space. We denote it further as $\mathbf{A}(\boldsymbol{x}) = \mathbf{A}$. We only consider 1D space here. Hence, $\nabla^2 = \partial_x^2$, $\mathbf{x} = x$ and $\mathbf{y} = y$. In 2D, front dynamics will be influenced by front curvature but this is minimal for the spatial scales considered [19].

Local rates of change

Here, we show how the local rates of change J_Y for Y any of the cover types (S, T, F, G) are chosen. As in any population model, we have

$$\text{change} \quad = \quad \text{gain} - \text{loss}$$

Below, the set of gain processes $\mathcal{P}_G$ contains recruitment and growth, while that of the loss processes $\mathcal{P}_L$ contains mortality from competition for resources, drought, fire and human impact. Each of those processes can be captured with a different term, such that the equations of the cover types S, T, F and G take the form

$$\dot{Y} = J_Y(\mathbf{Y}; \mathbf{A}) = \sum_{i \in \mathcal{P}_G} G_{Y,i}(\mathbf{Y}; \mathbf{A}) - \sum_{i \in \mathcal{P}_L} L_{Y,i}(\mathbf{Y}; \mathbf{A}, z).$$

As external climatic/edaphic forcing we choose

$$\mathbf{A} = (MAR, MSI, EFS),$$

where MAR stands for the observed multi-annual mean of rainfall, MSI Markham's seasonality index and EFS the edaphic suitability for forest [this lets our model agree with that of [8] but using a more compact notation]. Y refers to any of the cover types. Functions $G_{Y,i}$ and $L_{Y,i}$ are respectively, total gains and total losses per time of species Y by process i. The functional forms will be chosen inspired by an understanding of the effect of all relevant processes.

Gain functions consist of

$$\sum_{i \in \mathcal{P}_G} G_{Y,i}(\mathbf{Y}; \mathbf{A}) \quad = \quad \underset{\text{expansion,}}{G_{Y,e}(\mathbf{Y}; \mathbf{A})} + \underset{\text{recruitment,}}{G_{Y,r}(\mathbf{Y}; \mathbf{A})} + \underset{\text{mortality other types}}{G_{Y,m}(\mathbf{Y}; \mathbf{A})},$$

Gains can occur due to local (subgrid) expansion of vegetation ($i = e$), or, in case of an age-structured species, due to recruitment from a younger stage ($i = r$), or due to increased availability of space after mortality of other cover types ($i = m$). Loss functions consist of

$$\sum_{i \in \mathcal{P}_L} L_{Y,i}(\mathbf{Y};\mathbf{A}) = L_{Y,r}(\mathbf{Y};\mathbf{A}) + L_{Y,c}(\mathbf{Y};\mathbf{A}) + L_{Y,d}(\mathbf{Y};\mathbf{A}) + L_{Y,f}(\mathbf{Y};\mathbf{A}) + L_{Y,h}(\mathbf{Y},z) + L_{Y,o}(\mathbf{Y})$$

recruitment, competition, drought, fire, humans, other

They can occur due to: recruitment to an older stage ($i = r$), interspecific competition ($i = c$), drought ($i = d$), human impact ($i = h$) or other causes ($i = o$). Below, each of the gain/loss terms are discussed.

- *Cover gains and losses due to expansion.* Gains due to expansion involve increase of cover area of a more competitive cover type at cost of the cover area of a less competitive type. Hence,

$$G_{Y,e}(\mathbf{Y};\mathbf{A}) = R_Y(\mathbf{A})Y\mathbf{Y}.\mathbf{v}_{c,Y},$$

with Y the species that expands its cover and $\mathbf{v}_{c,Y}$ the competitiveness vector for cover type Y, which has ones at its elements where the corresponding cover type is less competitive than, and can be colonized by, Y. The rationale is that colonization by a species occurs due to an interaction of the space that is occupied by that species with the space that can be colonized by it. Having chosen the competitive hierarchy $F > T > S > G$ [10] in absence of water limitation, we have

$$\begin{aligned} G_{F,e}(\mathbf{Y};\mathbf{A}) &= R_S(\mathbf{A})T\mathbf{Y}.\mathbf{v}_{c,S} = R_F(\mathbf{A})F(G+S+T), \\ G_{S,e}(\mathbf{Y};\mathbf{A}) &= R_F(\mathbf{A})F\mathbf{Y}.\mathbf{v}_{c,F} = R_S(\mathbf{A})TG, \end{aligned}$$

These are removed from the areas of the less competitive types. Hence, losses due to expansion of competing types are

$$\begin{aligned} L_{T,c}(\mathbf{Y};\mathbf{A}) &= R_F(\mathbf{A})TF \\ L_{S,c}(\mathbf{Y};\mathbf{A}) &= R_F(\mathbf{A})SF \\ L_{G,c}(\mathbf{Y};\mathbf{A}) &= R_F(\mathbf{A})GF + R_T(\mathbf{A})GT \end{aligned}$$

The only age-structured part of our model is that of savanna saplings S and

savanna (adult) trees T. Note therefore that $G_{S,e}$ has TG instead of SG because expansion only comes from interaction of adult species T with places that saplings S can colonize (G). The dependence of R_Y on $\mathbf{A}$ captures the effect of water availability on growth, which we choose as

$$R_Y(\mathbf{A}) = \max[0, r_Y(1 - e^{-\mathbf{k_{R_Y}} \cdot \mathbf{A} + a_Y})],$$

with r_Y the maximal growth rate, $\mathbf{k_{R_Y}}$ as growth rate increase for every component of $\mathbf{A}$ and a_Y fixing the rate for $\mathbf{A} = \mathbf{0}$. This function captures saturation of growth rate where water limitation is less severe, which is supported by empirical work (e.g. saturation of NDVI as a function of rainfall and high temporal correlations between rainfall and NDVI below saturation; [35, 36]).

- *Gains and losses due to recruitment.* Saplings will recruit into adults, such that,

$$G_{T,r}(\mathbf{Y}; \mathbf{A}) = -L_{S,r}(\mathbf{Y}; \mathbf{A}) = Q(\Phi)S,$$

where recruitment rate Q is a function of burnt area fraction Φ,

$$Q(\Phi) = Q_0(1 - h\Phi).$$

Q_0 is the recruitment rate in absence of fire and $Q_0(1-h)$ the recruitment rate in presence of fire for a particular year, with $0 \leq h \leq 1$. Hence, in agreement with previous empirical work, fire affects the establishment rather than the mortality of savanna trees.

- *Base mortality.* This is the base mortality in absence of fire, drought, competition or human impact. As is common in ecology, we choose this mortality linear in Y,

$$L_{Y,o} = m_{Y,o}Y$$

with $m_{Y,o}$ the base mortality rate of Y.

- *Losses due to drought.* Drought-related mortality rate $M_Y(\mathbf{A})$ will be chosen such that drought effects cause increased mortality below a certain threshold of $\mathbf{A}$. We

choose

$$L_{Y,d}(Y;\mathbf{A}) = M_Y(\mathbf{A})Y,$$

with

$$M_Y(\mathbf{A}) = e^{-\mathbf{k_{M_Y}}\cdot\mathbf{A}+a_{M_Y}},$$

or when absorbing the base mortality rate into this term,

$$M_Y(\mathbf{A}) = m_{Y,o} + e^{-\mathbf{k}_{M_Y}\cdot\mathbf{A}+a_{M_Y}}.$$

Such nonlinear increase of mortality with dryness is assumed to be a consequence of exceedance of thresholds related to tree water availability.

- *Losses due to fire.* Φ is burnt area fraction per year. Grasses and savanna tree saplings resprout very rapidly so they are assumed to be unaffected by fire on the considered time scale. Savanna trees are fire adapted so also they are also assumed to be unaffected by fire [10]. Hence, in this model, only forest trees experience direct mortality due to fire. This mortality is chosen proportional to burnt area and burning is assumed to occur in a homogeneously distributed fashion over the pixel. Thus

$$L_{F,f}(\mathbf{Y};\mathbf{A}) = b\Phi F,$$

where b is the fraction of fire affected forest that dies, also the fire sensitivity of forest cover. Previous research shows that fire in the tropics is determined by climate on large scales and by tree cover on small scales [4, and the references therein]. On the other hand, local-scale forest distribution affects fire occurrence. This means that forest and fire interact on local scales. Hence, a positive feedback can arise if this interaction occurs in a nonlinear fashion and if it reinforces changes. There exists evidence from independent lines of research for such positive feedbacks [4]. A fire feedback operating on small spatial scales is crucial for producing the two stable states, savanna and forest [7, 10, 37]. We parameterized the fire feedback by choosing burnt area fraction $\mathbb{F}$ as a sigmoid-shaped function of fire-prone cover, consistent with fire percolation models. We let this function

further also depend on climate. The double-striked notation is used to distinguish it from the functions and variables related to the cover types. The fundamental process responsible for the fire feedback is small-scale spatial fire percolation over a fire prone layer. Simulations have shown [37] that this process induces a sharp increase of fire-related mortality around the percolation threshold, which occurs when about 60% of the landscape is fire-prone. We do not intend to model this percolation process but take a mesoscale approach, where we choose $\Phi(Y;\mathbf{A})$ to have a sharp increase around a total nonherbaceous cover $1-G-S=T+F=Y_c$. Instead of modeling a positive threshold response of fire on fire-conductive cover $(S+G)$, we choose to formulate the functional form as a negative threshold response of fire on non-fire-conductive cover $(T+F)$. This makes the analysis easier while keeping the model qualitatively the same. We hence choose

$$\Phi(T,F;\mathbf{A}) = \frac{1}{\tau}\frac{Y_c(\mathbf{A})^n}{Y_c(\mathbf{A})^n+(T+F)^n}. \tag{S1}$$

The exponent n is a positive integer that controls the steepness of increase of burnt area fraction at $Y \approx Y_c$. We chose $n=4$. $Y_c(\mathbf{A})$ captures the varying percolation threshold with hydrological conditions - a lower threshold in drier environments. This dependence is chosen to be piecewise linear,

$$Y_c(\mathbf{A}) = \max[0, Y_{c,0} + \mathbf{k}_c.\mathbf{A}].$$

Here, $\mathbf{k}_c$ is a constant vector and $Y_{c,0}$ a constant scalar. The elements of $\mathbf{k}_c$ represent the sensitivity of $Y_c(\mathbf{A})$ to the different components of $\mathbf{A}$. Y_c has a value of about 40% for common conditions, which is the tree cover value at which fire has been observed to increase [8, 38, 39].

- *Losses due to human impact.* Deforestation of forest trees is chosen as

$$L_{F,h}(F,z) = C(z)F,$$

where $C(z)$ is the deforestation rate and z distance from anthropogenically

impacted areas. We choose

$$C(z) = ce^{-k_C z},$$

such that the deforestation rate decays with distance from impacted areas. c is the the maximum deforestation rate, which occurs in agricultural areas ($z = 0$).

- *Gains due to mortality of other cover types.* When any cover type loses space, it makes place for other cover types. When this does not occur due to competition or recruitment, grass is the default cover type that gains ground. This agrees with taking the assumption that grass grows back instantly, which was also taken in [10]. Therefore,

$$G_{G,m}(\mathbf{Y};\mathbf{A}) = \sum_{\substack{Y \in \{S,T,F\} \\ i \in \{d,f,h,o\}}} L_{Y,i}(\mathbf{Y};\mathbf{A}),$$

of which the terms are defined above.

The system of equations locally obeys the aforementioned mathematical constraint for every point in time

$$\sum_{Y \in \{S,T,F,G\}} Y = 1.$$

Differentiation of the above equation with respect to time yields

$$\begin{aligned} \sum_{Y \in \{S,T,F,G\}} \partial_t Y &= 0, \\ \sum_{Y \in \{S,T,F,G\}} J_Y(\mathbf{Y};\mathbf{A}) &= 0. \end{aligned} \tag{S2}$$

The conservation equation S2 implies that the sum of all loss and gain terms should be zero. We can see that this is the case because: (i) total expansion due to successful competition is at the cost of total loss due to unsuccessful competition, (ii) recruitment lost by S is gained by T, (iii) tree cover losses due to fire, drought and human impact are gained by grass cover.

Spatial dependence

Thus far, we have only treated the dynamics as spatially independent $[\mathbf{Y} = \mathbf{Y}(t)]$, for particular parameter values of hydrology $\mathbf{A}$ and distance to human impacted areas z. To run this model for a whole region $[\mathbf{Y} = \mathbf{Y}(\mathbf{x}, t)]$, we need to take into account not only the spatial heterogeneity of these variables but also the relevant spatial interactions.

1. *Spatial heterogeneity.* Climatic, edaphic and anthropogenic spatial heterogeneity can be included by taking $\mathbf{A}$ and z as functions of space $\mathbf{A}(\mathbf{x})$ and $z(\mathbf{x})$.

2. *Spatial interaction.* We assume that diffusion of cover types only occurs due to spread of seeds. We do not model seed dispersal but approximate it by dispersal of saplings. Hence the diffusion coefficient of savanna adult tree cover is zero. That of forest cover is not zero because part of its population is in the sapling stage. Hence,

$$\mathbf{D} = (D_S, 0, D_F, 0).$$

The cover types that diffuse from neighboring areas settle in the areas that are taken by grasses. Therefore, in the spatial model, the diffusion terms are also grass cover loss terms due to unsuccessful competition, or

$$L_{G,c}(\mathbf{Y}; \mathbf{A}) = R_F(\mathbf{A})GF + R_T(\mathbf{A})GT + D_S\nabla^2 S + D_F\nabla^2 F.$$

Spatial interaction can also occur due to spread of fire. While analysis of the model with fire spread is a bit more involved [19], the conclusions are the same.

Forest-savanna model

Here, we develop the forest-savanna model with all previously mentioned cover types. Note that we do not write the explicit dependence on space and time. Hence, this means that all cover types are a function of space $\mathbf{x}$ and time t. The forcings are only a

function of space, i.e. $\mathbf{A} = \mathbf{A}(\mathbf{x})$ and $z = z(\mathbf{x})$. Based on the previous sections, we have

$$\begin{aligned}
\partial_t S &= G_{S,e}(\mathbf{Y};\mathbf{A}) - L_{S,r}(\mathbf{Y};\mathbf{A}) - L_{S,d}(S;\mathbf{A}) - L_{S,c}(\mathbf{Y};\mathbf{A}) + D_S \nabla^2 S,\\
\partial_t T &= G_{T,r}(\mathbf{Y};\mathbf{A}) - L_{T,d}(T;\mathbf{A}) - L_{T,c}(\mathbf{Y};\mathbf{A}),\\
\partial_t F &= G_{F,e}(\mathbf{Y};\mathbf{A}) - L_{F,d}(F;\mathbf{A}) - L_{F,f}(\mathbf{Y};\mathbf{A}) - L_{F,h}(F,z) + D_F \nabla^2 F,\\
\partial_t G &= -L_{G,c}(\mathbf{Y};\mathbf{A}) + G_{G,m}(\mathbf{Y};\mathbf{A}),
\end{aligned}$$

Filling in the gains and losses and making use of $G = 1 - S - T - F$, we obtain

$$\begin{aligned}
\partial_t S &= R_s(\mathbf{A})(1 - S - T - F)T - Q[\Phi(T, F;\mathbf{A})]S - M_S(\mathbf{A})S - R_F(\mathbf{A})SF,\\
\partial_t T &= Q(\Phi)S - M_T(\mathbf{A})T - R_F(\mathbf{A})TF,\\
\partial_t F &= R_F(\mathbf{A})(1 - F)F - b\Phi(T, F;\mathbf{A})F - M_F(\mathbf{A})F - C(z)F + D_F \nabla^2 F,
\end{aligned}$$

We briefly remind the reader of some of the model parameters shown here. This model is forced by the spatial distribution of $\mathbf{A}$ and the distance to human impact z. b is the (constant) sensitivity of forest cover to fire. Q represents sapling recruitment into adults and is a linearly decreasing function of burnt area fraction. $C(z)$ is the deforestation rate which decays with z. Note that the simulation model used to produce Fig 1A has no deforestation term in the equation of $\partial_t T$. When hiding the dependence on $\mathbf{A}$, we have

$$\begin{aligned}
\partial_t S &= R_s(1 - S - T - F)T - Q[\Phi(T, F)]S - M_S S - R_F SF + D_S \nabla^2 S,\\
\partial_t T &= Q[\Phi(T, F)]S - M_T T - R_F TF,\\
\partial_t F &= R_F(1 - F)F - b\Phi(T, F)F - M_F F - C(z)F + D_F \nabla^2 F, \qquad \text{(S3)}
\end{aligned}$$

Forest growth rate r_F

Here, we will show how we derived the maximum forest growth rate. We do this to set the time scale of the model dynamics (other parameters were initially estimated relative to r_F). The steady state forest cover value under sufficiently moist conditions is about 80%. Therefore, we made sure that this also occurs in the model by first seeing that in moist conditions far from human-impacted areas, $C_F = 0$, $\Phi = 0$, $R_F = r_F$ and

$M_F = m_{F,o}$ such that

$$\frac{dF}{dt} = r_F(1-F)F - m_{F,o}F.$$

We will further set $m_{F,o} \equiv m$ and $r_F \equiv r$. The ODE can be solved by separation of variables using partial fractions such that the time that forest needs to grow from F_0 to $F_1 > F_0$ is

$$t_1 - t_0 = \frac{1}{r(1-m)} \log(\frac{|1-m-F_0|\,F_1}{|1-m-F_1|\,F_0}). \tag{S4}$$

If we take as initial tree cover a small value that could result from noise and as final tree cover the carrying capacity, we have

$$F_0 = 0.01,\ F_1 = 0.8.$$

At carrying capacity, we have $r(1-F)F - mF = 0$,such that

$$F^* = 1 - \frac{m}{r},\ F^* = 0.$$

of which only the first is stable. As the data shows that F^* has to be equal to 0.8, such that $m = 0.2r$. Substituting this and the values for F_0, F_1, we obtain

$$t_1 - t_0 = \frac{1}{r(1-0.2r)} \log(400\frac{0.99-0.2r}{1-r}). \tag{S5}$$

This function is plotted in S2 Fig. In a recent study on recovery of secondary forests [40], it was found that moist forests regain the median value of old-growth forests after about at least 30 years. Therefore, we chose the r value consistent with this time, which is

$$r \approx 0.2.$$

Literature Cited

1. Townshend JRG, Carroll ML, DiMiceli CM, Sohlberg RA, Hansen MC, DeFries R. type [; 2011]Available from: https://lpdaac.usgs.gov/dataset_discovery/modis/modis_products_table/mod44b.

2. Hirota M, Holmgren M, van Nes EH, Scheffer M. Global Resilience of Tropical Forest and Savanna to Critical Transitions. Science. 2011;334(6053):232–235. doi:10.1126/science.1210657.

3. Staver AC, Archibald S, Levin SA. The Global Extent and Determinants of Savanna and Forest as Alternative Biome States. Science. 2011;334(6053):230–232. doi:10.1126/science.1210465.

4. Pausas JG, Dantas VdL. Scale matters: fire–vegetation feedbacks are needed to explain tropical tree cover at the local scale. Global Ecology and Biogeography. 2017;26(4):395–399. doi:10.1111/geb.12562.

5. Ratajczak Z, Nippert JB. Comment on "Global Resilience of Tropical Forest and Savanna to Critical Transitions". Science. 2012;336(6081).

6. Good P, Harper A, Meesters A, Robertson E, Betts R. Are strong fire-vegetation feedbacks needed to explain the spatial distribution of tropical tree cover? Global Ecology and Biogeography. 2016;25(1):16–25. doi:10.1111/geb.12380.

7. van Nes EH, Hirota M, Holmgren M, Scheffer M. Tipping points in tropical tree cover: linking theory to data. Global Change Biology. 2014;20(3):1016–1021. doi:10.1111/gcb.12398.

8. Wuyts B, Champneys AR, House JI. Amazonian forest-savanna bistability and human impact. Nature Communications. 2017;8(May):15519. doi:10.1038/ncomms15519.

9. Staal A, Dekker SC, Xu C, van Nes EH. Bistability, Spatial Interaction, and the Distribution of Tropical Forests and Savannas. Ecosystems. 2016;19(6):1080–1091.

10. Staver AC, Levin SA. Integrating theoretical climate and fire effects on savanna and forest systems. The American Naturalist. 2012;180(2):211–224.

11. van Nes EH, Staal A, Hantson S, Holmgren M, Pueyo S, Bernardi RE, et al. Fire forbids fifty-fifty forest. PloS One. 2018;13(1):e0191027. doi:10.1371/journal.pone.0191027.

12. Van De Leemput IA, van Nes EH, Scheffer M. Resilience of alternative states in spatially extended ecosystems. PloS one. 2015;10(2):1–17. doi:10.1371/journal.pone.0116859.

13. Meron E. Nonlinear physics of ecosystems. CRC Press; 2015.

14. Murray JD. Mathematical biology. I: An Introduction, volume 17 of Interdisciplinary Applied Mathematics. Springer-Verlag, New York; 2002.

15. Huang K. Introduction to statistical physics. CRC press; 2009.

16. Touboul JD, Staver AC, Levin SA. On the complex dynamics of savanna landscapes. Proceedings of the National Academy of Sciences. 2018; p. 201712356. doi:10.1073/pnas.1712356115.

17. MathWorks. type [; 2012]Available from: `uk.mathworks.com`.

18. Wolfram Research Inc. type [; 2018].

19. Wuyts B, Champneys AR, House JI. Bistability of Tropical Vegetation in a Heterogeneous Environment [PhD thesis]. University of Bristol; 2017.

20. Pismen LM. Patterns and interfaces in dissipative dynamics. Springer Science & Business Media; 2006.

21. Avitabile D. Numerical calculation of coherent structures in spatially-extended neural networks; 2016. Available from: `https://icmns2016.inria.fr/pre-conference-workshop/`.

22. Rankin J, Avitabile D, Baladron J, Faye G, Lloyd DJB. Continuation of localised coherent structures in nonlocal neural field equations. SIAM Journal on Scientific Computing. 2013;36(1):B70–B93. doi:10.1137/130918721.

23. Beyn WJ, Champneys A, Doedel E, Govaerts W. Numerical continuation, and computation of normal forms. Handbook of Dynamical Systems. 2000;.

24. Sherratt JA, Lewis MA, Fowler AC. Ecological chaos in the wake of invasion. Proceedings of the National Academy of Sciences of the United States of America. 1995;92(7):2524–8.

25. Sherratt JA, Smith MJ. Periodic travelling waves in cyclic populations: field studies and reaction-diffusion models. Journal of the Royal Society, Interface. 2008;5(22):483–505. doi:10.1098/rsif.2007.1327.

26. Strogatz SH. Nonlinear dynamics and chaos: with applications to physics, biology, chemistry, and engineering. Westview press; 2014.

27. Grainger A. Constraints on modelling the deforestation and degradation of tropical open woodlands; 1999. Available from: http://www.jstor.org/stable/2997881.

28. Hanan NP, Tredennick AT, Prihodko L, Bucini G, Dohn J. Analysis of stable states in global savannas: is the CART pulling the horse? Global Ecology and Biogeography. 2014;23(3):259–263. doi:10.1111/geb.12122.

29. Gerard F, Hooftman D, van Langevelde F, Veenendaal E, White SM, Lloyd J. MODIS VCF should not be used to detect discontinuities in tree cover due to binning bias. A comment on Hanan et al. (2014) and Staver and Hansen (2015). Global Ecology and Biogeography. 2017;26(7):854–859. doi:10.1111/geb.12592.

30. Paiva AO, Silva LCR, Haridasan M. Productivity-efficiency tradeoffs in tropical gallery forest-savanna transitions: linking plant and soil processes through litter input and composition. Plant Ecology. 2015;216(6):775–787. doi:10.1007/s11258-015-0466-8.

31. Eltahir EAB, Bras RL. Precipitation recycling in the Amazon basin. Quarterly Journal of the Royal Meteorological Society. 1994;120(518):861–880. doi:10.1002/qj.49712051806.

32. Pielke RA. Influence of the spatial distribution of vegetation and soils on the prediction of cumulus Convective rainfall. Reviews of Geophysics. 2001;39(2):151–177. doi:10.1029/1999RG000072.

33. Pöschl U, Martin ST, Sinha B, Chen Q, Gunthe SS, Huffman JA, et al. Rainforest aerosols as biogenic nuclei of clouds and precipitation in the Amazon. Science (New York, NY). 2010;329(5998):1513–6. doi:10.1126/science.1191056.

34. Zemp DC, Schleussner CF, Barbosa HMJ, Hirota M, Montade V, Sampaio G, et al. Self-amplified Amazon forest loss due to vegetation-atmosphere feedbacks. Nature Communications. 2017;8:14681. doi:10.1038/ncomms14681.

35. Scheffer M, Holmgren M, Brovkin V, Claussen M. Synergy between small-and large-scale feedbacks of vegetation on the water cycle. Global Change Biology. 2005;11(7):1003–1012. doi:10.1111/j.1365-2486.2005.00962.x.

36. Wuyts B, Govers G. A System Perspective on Sahel Greening [MSc Thesis]. University of Leuven; 2012.

37. Schertzer E, Staver AC, Levin SA. Implications of the spatial dynamics of fire spread for the bistability of savanna and forest. Journal of Mathematical Biology. 2015;70(1-2):329–341. doi:10.1007/s00285-014-0757-z.

38. Archibald S, Roy DP, van Wilgen BW, Scholes RJ. What limits fire? An examination of drivers of burnt area in Southern Africa. Global Change Biology. 2009;15(3):613–630. doi:10.1111/j.1365-2486.2008.01754.x.

39. Staver AC, Archibald S, Levin S. Tree cover in sub-Saharan Africa: Rainfall and fire constrain forest and savanna as alternative stable states. Ecology. 2011;92(5):1063–1072. doi:10.1890/i0012-9658-92-5-1063.

40. Poorter L, Bongers F, Aide TM. Biomass Resilience of Neotropical Secondary Forests. Nature. 2016;530(7589):211–227.
